# Seeking the views of service users: From impossibility to necessity

**DOI:** 10.1111/hex.12621

**Published:** 2017-09-15

**Authors:** Louise Condon

**Affiliations:** ^1^ College of Human and Health Sciences Swansea University Wales UK

When I was a graduate student nurse in the early 1980s, one assessment was to devise a research project—I suggested asking patients for their views on the care they had received in hospital. Sadly, when I proposed this topic, I was told that asking patients for their views was far too risky and certainly could not be carried out in my training hospital. Imagine my delight therefore at being an editor of Health Expectations journal which seeks to represent and explore the views of service users and the ways in which they can be involved in research and service development. Evaluating patients’ experiences is now considered a vital component of assessing the quality of health care,^1^, ^2^ and in this edition Smirnova et al. report on their validation of the Consumer Quality Index (CQI) questionnaire as a standardized measure for evaluating inpatient experiences in Dutch hospitals. A limitation in using questionnaires to measure patient experience is that survey response rates are notoriously low, and the authors recommend exploring reasons for non‐response to ensure an adequate sample size and optimal use of resources.

This edition of HEX presents a variety of studies in which service user voice adds to our understanding of how we can provide better health services. Communication with patients is the theme of four papers. Chevalier et al. used communication accommodation theory (CAT) to improve communication between pharmacists and patients to avoid medication‐related problems (MRPs). In accommodative communication, speakers complement their speech patterns (e.g. rate, volume, tone), modulate language and word use to maximize understanding, take turns and respond to non‐verbal cues. In this study, most pharmacists used CAT strategies, but non‐accommodation occurred when pharmacists spoke too quickly, used unfamiliar terms and did not involve patients in agenda setting. Chou et al. analysed oncologists’ discussion of prognosis with African American patients diagnosed with cancer; their findings highlight the need to improve clinical communication with people from minority ethnic backgrounds who have advanced cancer. In a mixed methods study, Eassey et al. explored the MRPs experienced by Australian patients (n = 506) on discharge from hospital. A third of respondents experienced problems such as unwanted side‐effects and being given unfamiliar medication which resulted in confusion and anxiety. Patients would have liked more information about the medications they were prescribed and emphasized the need for better collaboration and communication (“I have nine specialists, they need to swap notes!”). Finally, communication emerged as a priority in Lawn et al.'s investigation into ‘what cancer survivors want?’ When care is shared between hospital specialists and primary care participants want to be at the centre of care, for instance, having shared electronic health records, and to be prepared for self‐management.

Shared decision making (SDM) is central to patient‐centred care and closely related to the protection of patients’ rights. A cluster‐randomized trial of SDM in patients with type 2 diabetes (Ouden, Vos and Rutten) gave strong indications that SDM improved treatment outcomes, while Perestelo‐Perez et al.'s randomized trial of the effectiveness of a decision aid for patients with depression showed improved knowledge and reduced decisional conflict. In a qualitative study of patients with a newly acquired spinal cord injury, Scheel‐Sailer et al. identified a challenge for health professionals in balancing patients’ need for autonomy with their reduced ability to participate in decision making in the early months post‐injury. The authors suggest providing adequate information is an important way to address this conflict. Health information is the topic of studies by Cusack et al., Learmouth et al. and Brady et al. McKenna et al. stress the importance of context (e.g., quality of communication with the health‐care provider, family medical history) in understanding service users’ health literacy skills.

A group of papers explore service development and service users’ views on “doing things differently.” Imison^3^ commented in 2009 that, despite the skills and employment challenges facing the health sector, traditional patterns of working and service provision have changed little in the UK since the inception of the NHS in 1948. The physician associate (PA) is a new role developed in the United States in the 1960s to provide clinical services which may be a substitute for, or delegated, from doctors, and now introduced in the UK. In a qualitative study, Halter et al. found that as with nurse substitutes for doctors, trust is a crucial factor, and that patients responded more favourably to PAs when the role was clearly explained to them. Willingness to consult a PA again was linked to patients’ opinions on the severity of their condition, as well as to experience of care and treatment. Two studies focused on service users’ roles in caring for themselves. Williams et al. explored women's perspectives on self‐sampling for HPV (human papillomavirus), as this test is likely to be introduced into the UK cervical screening programme, with self‐sampling a possibility. Self‐management of type 2 diabetes (such as taking medication, eating well and taking exercise, caring for feet and attending health checks) is a vital part of keeping healthy, but people with severe mental illness experience more difficulty with this; Mulligan et al. suggest where new interventions could be targeted.

The diverse range of topics and approaches presented in this edition of Health Expectations illustrates what a distance has been travelled since the 1980s in valuing the opinions of service users and recognizing the validity of their contribution to research and health service development.
